# Valorizing Grape Pomace: A Review of Applications, Nutritional Benefits, and Potential in Functional Food Development

**DOI:** 10.3390/foods13244169

**Published:** 2024-12-23

**Authors:** Vladimir S. Kurćubić, Nikola Stanišić, Slaviša B. Stajić, Marko Dmitrić, Saša Živković, Luka V. Kurćubić, Vladimir Živković, Vladimir Jakovljević, Pavle Z. Mašković, Jelena Mašković

**Affiliations:** 1 Department of Food Technology, Faculty of Agronomy, University of Kragujevac, Cara Dušana 34, 32102 Čačak, Serbia; 2 Institute for Animal Husbandry, Belgrade-Zemun, Autoput Beograd-Zagreb 16, 11000 Belgrade, Serbia; nikola0135@yahoo.com; 3 Department of Animal Products Technology, Faculty of Agriculture, University of Belgrade, Nemanjina 6, 11080 Belgrade, Serbia; stajic@agrif.bg.ac.rs; 4 Veterinary Specialized Institute “Kraljevo”, Žička 34, 36000 Kraljevo, Serbia; markodmitric@gmail.com (M.D.); sasha98@live.se (S.Ž.); 5 Department of Medical Microbiology, University Clinical Center of Serbia, Pasterova 2, 11000 Beograd, Serbia; kurcubiclk@gmail.com; 6 Department of Physiology, Faculty of Medical Sciences, University of Kragujevac, 69 Svetozara Markovica St., 34000 Kragujevac, Serbia; vladimirziv@gmail.com (V.Ž.); drvladakgbg@yahoo.com (V.J.); 7 Department of Human Pathology, Sechenov First Moscow State Medical University, 8 Trubetskaya St., 119991 Moscow, Russia; 8 Department of Chemistry and Chemical Engineering, Faculty of Agronomy, University of Kragujevac, Cara Dušana 34, 32102 Čačak, Serbia; pavlem@kg.ac.rs (P.Z.M.); jelenav@kg.ac.rs (J.M.)

**Keywords:** grape pomace, functional food, nutraceuticals, antioxidative effects, antimicrobial activity, circular economy

## Abstract

Grape pomace (GP), a byproduct of winemaking, has gained significant attention as a sustainable and functional ingredient with applications in the food and nutraceutical industries. This review examines the potential of GP in meat products and analogs, functional foods, and nutraceuticals, highlighting its composition, health benefits, and role in enhancing nutritional and functional properties. Rich in dietary fiber, polyphenols, essential fatty acids, and bioactive compounds, GP exhibits antioxidant, anti-inflammatory, and gut health-promoting effects, making it suitable for various food applications. Its incorporation into meat products, such as sausages and patties, improves texture, enhances shelf life, and increases nutritional value while reducing the environmental footprint. GP is also effective in functional foods such as baked goods, dairy and plant-based yoghurts, smoothies, and snack bars, where it can enrich fiber and polyphenol content, aid in satiety, and provide health benefits beyond basic nutrition. The challenge is how to maintain the sensory properties characteristic of conventional, unmodified products. In nutraceuticals, GP’s polyphenolic compounds and dietary fiber support antioxidant, anti-inflammatory, and metabolic health functions, with applications as antioxidant supplements, gut health boosters, weight management aids, and cardiovascular health supplements. Despite challenges such as taste modification and optimizing bioavailability, GP’s versatility and sustainability highlight its value in developing innovative, health-oriented products. This review emphasizes the promise of GP as a valuable ingredient in functional foods and nutraceutical formulations, contributing to health, sustainability, and resource efficiency.

## 1. Introduction

The global food industry faces the critical challenge of balancing sustainability with nutritional advancement, especially as populations grow and environmental pressures escalate. A significant aspect of this challenge is managing food industry byproducts, which contribute substantially to global waste yet possess untapped nutritional and functional potential. One prominent sector, wine production, generates large quantities of grape pomace (GP)—the byproduct of grape skins, seeds, and stems left after juice extraction.

Historically, GP has either been discarded or used for composting. However, recent studies highlight its value as a rich source of dietary fiber (DF), polyphenols, minerals, and other bioactive compounds. This emerging awareness presents a unique opportunity to repurpose GP as an ingredient of functional foods, nutraceuticals, and meat analogs, thereby supporting both environmental sustainability and human health [1,2]. Incorporating GP into food products aligns with the broader shift towards circular economy principles, wherein waste materials are recycled back into production cycles. This approach maximizes resource efficiency and minimizes environmental impact. The food industry benefits significantly from such an approach, as utilizing byproducts like GP reduces waste and meets consumer demand for healthier, more sustainable products. GP’s high dietary fiber content (50–75% of its dry matter), along with substantial amounts of protein, healthy fats, and polyphenols, makes it particularly suitable for creating functional foods and nutraceuticals that cater to a health-conscious market [3].

The polyphenolic compounds in GP—such as catechins, proanthocyanidins, and flavonols—are well known for their antioxidant, anti-inflammatory, and antimicrobial effects, which contribute to the prevention of various chronic diseases and enhance overall health [1,4]. Beyond its health benefits, GP has functional properties that make it a valuable addition to a wide range of food products. For example, its fiber and polyphenol content can improve foods’ texture and sensory qualities, making it ideal for incorporation into baked goods, dairy products, beverages, and meat analogs [5,6]. Dietary fibers from GP contribute to water retention, structure, and flavor stability in processed foods, while polyphenols enhance antioxidant capacity, potentially extending shelf life [6]. These attributes are especially relevant in developing meat substitutes and low-fat or reduced-salt products, where GP can improve texture and palatability without sacrificing nutritional quality [5].

The concept of functional foods and nutraceuticals has garnered significant attention in recent years as consumers increasingly seek products that provide health benefits beyond basic nutrition. Functional foods are defined as those that offer specific health advantages through their naturally occurring compounds—such as improved digestion, reduced cholesterol levels, and enhanced immune function. On the other hand, nutraceuticals refer to bioactive compounds extracted and concentrated into supplements for health promotion and disease prevention. GP fits well into both categories, offering high concentrations of bioactive compounds that support metabolic health, cardiovascular function, and gut microbiota balance [3].

The potential to incorporate GP into various food matrices—including bakery products, dairy alternatives, and meat analogs—positions it as an attractive ingredient in the rapidly growing market for functional foods and nutraceuticals [5,7]. Moreover, applying GP in meat and meat analog products presents an innovative strategy to enhance the health profile of these foods while meeting the growing demand for plant-based alternatives. Although meat products are widely consumed, they are often criticized for their high saturated fat and salt content, as well as their low fiber levels—factors linked to increased risks of cardiovascular disease, obesity, and other health issues. Incorporating GP adds valuable dietary fiber and polyphenols and helps reduce the need for fat and artificial additives, aligning with consumer preferences for clean-label products [8].

This review aims to evaluate grape pomace’s nutritional and functional value in the food industry. By consolidating current research on the application of GP in various food products, this review seeks to provide insights into how this byproduct can be effectively utilized to meet consumer demands, promote sustainability, and enhance public health.

## 2. Nutritional Composition of Grape Pomace

Grape pomace (GP) is rich in dietary fiber, proteins, lipids and phenolic compounds, with the composition varying depending on grape variety and processing conditions [1,9]. A summary of GP’s nutritional composition is given in Table 1.

GP is a significant source of dietary fiber, containing approximately 50–60% fiber, primarily in insoluble forms like cellulose, hemicellulose, and lignin [11]. Insoluble fiber is beneficial for gut health as it promotes regular bowel movements and helps prevent constipation by increasing stool bulk and retaining water [12]. Moreover, dietary fiber is associated with a reduced risk of cardiovascular diseases, obesity, and type 2 diabetes due to its ability to regulate blood glucose levels and lower cholesterol absorption [13,14].

While present in smaller amounts, the soluble fiber in GP provides prebiotic benefits by nurturing beneficial gut bacteria. These bacteria ferment soluble fiber to produce short-chain fatty acids (SCFAs) such as butyrate, acetate, and propionate. SCFAs play essential roles in immune modulation, reduction in inflammatory markers, and protection against colon cancer [15,16].

The polyphenolic content in GP varies based on grape variety, processing conditions, and extraction methods. Polyphenols—including flavonoids, proanthocyanidins, and phenolic acids—contribute to the strong antioxidant properties of GP, making it valuable for health applications and as a natural preservative in foods [1,17]. These compounds have been linked to protective effects against oxidative stress and inflammation associated with ageing, cardiovascular diseases, cancer, and neurodegenerative conditions [18,19].

Proanthocyanidins, among the primary polyphenols found in GP, exhibit significant antioxidant activity. Research has shown their ability to scavenge free radicals and prevent lipid oxidation in food systems [20,21]. Additionally, flavonoids in GP have demonstrated potential in reducing the oxidation of low-density lipoprotein (LDL) cholesterol, which is important for cardiovascular protection [9,22].

Grape seeds within the pomace contain valuable essential fatty acids, particularly linoleic acid (an omega-6 fatty acid), vital for maintaining healthy cell membranes, skin integrity, and overall inflammatory balance. Linoleic acid is associated with cardiovascular health benefits, such as lowering blood pressure and improving lipid profiles [23,24]. Thus, these fatty acids contribute to GP’s appeal as a functional food ingredient with potential therapeutic applications [8].

Existing studies on the nutritional composition of GP, however, focus on compositional data without addressing the variability introduced by factors such as grape variety, processing methods, and seasonal changes [9]. For example, while Vazquez-Armenta et al. [1] present comprehensive data, their analysis is limited to specific cultivars, raising questions about generalizability to other grape varieties. Similarly, while studies like those by Dvoir et al. [10] provide valuable insights into the use of GP in various forms, they often lack information on how storage or drying processes may affect nutritional quality. Future research should standardize methodologies and expand datasets to ensure broader applicability.

## 3. Health Benefits of Grape Pomace

Grape pomace (GP) has demonstrated numerous health benefits, supported by empirical studies. The dietary fiber found in GP is primarily insoluble, which supports digestive health by increasing stool bulk, enhancing water retention, and promoting regular bowel movements. These benefits are essential for preventing constipation and maintaining colon health [12,25]. Additionally, the fiber in GP can act as a prebiotic, fostering the growth of beneficial gut bacteria, such as *Bifidobacterium* and *Lactobacillus* [26], and increasing the production of short-chain fatty acids (SCFAs) in the colon. SCFAs are advantageous for immune health and may reduce the risk of colon cancer [15,16].

GP is also rich in polyphenols, including flavonoids, proanthocyanidins, and phenolic acids, which provide significant antioxidant and anti-inflammatory benefits. These compounds protect against oxidative stress by scavenging free radicals, reducing the risk of chronic diseases such as cardiovascular disease, cancer, and neurodegeneration [1,18]. In particular, flavonoids in GP prevent the oxidation of LDL cholesterol, which can lower the risk of atherosclerosis [17,22]. Mechanistically, these effects are mediated by the modulation of endothelial nitric oxide synthase (eNOS) and inhibition of oxidative stress pathways [27] (Li et al., 2022).

The fiber and polyphenols in GP may also assist with weight management and metabolic health. They enhance feelings of fullness, reduce spikes in blood glucose levels, and lower cholesterol absorption [28,29]. Polyphenols improve insulin sensitivity and glucose metabolism, which may help reduce the risk of metabolic syndrome and type 2 diabetes [26].

Moreover, the antioxidants and essential fatty acids in GP contribute to cardiovascular health by improving vascular function, lowering blood pressure, and reducing inflammation [8,24]. GP may also lower blood lipid levels and prevent the oxidation of LDL cholesterol, resulting in a healthier lipid profile and a reduced risk of cardiovascular disease [23]. Emerging studies also suggest that GP polyphenols upregulate antioxidant defense enzymes, including superoxide dismutase (SOD) and glutathione peroxidase (GPx), providing cellular protection under oxidative stress conditions [30].

Finally, GP polyphenols, particularly proanthocyanidins, exhibit anticancer properties by inhibiting cancer cell proliferation, inducing apoptosis, and protecting against DNA damage [17,18]. The fiber content in GP also decreases colon cells’ exposure to carcinogens, reducing the risk of colorectal cancer [12,15].

It is worth noticing that most studies rely on in vitro models or animal studies, limiting their direct applicability to human health. For instance, Anderson et al. [13] demonstrate significant antioxidant effects of GP in controlled lab settings, but the absence of human clinical trials weakens the translational potential of these findings. Moreover, the bioavailability of phenolic compounds in humans remains a critical gap in the literature [14]. Empirical evidence from randomized clinical trials could substantiate these health claims more convincingly.

## 4. Functional Foods and Nutraceuticals

The emergence of functional foods and nutraceuticals has significantly changed the food industry by combining nutrition and health benefits in everyday diets. GP, a nutrient-rich byproduct of the wine industry, shows considerable potential due to its high levels of dietary fiber, polyphenols, essential fatty acids, and bioactive compounds. Functional foods are designed to offer health benefits beyond basic nutrition, and the composition of GP aligns with this objective.

This makes it suitable for developing new food products with additional health-promoting properties. However, the extent of its benefits depends on factors such as bioavailability, processing methods, and the scalability of its incorporation into diverse food systems. This section examines the functional components of GP and its various applications in nutraceutical formulations.

### 4.1. Nutritional and Bioactive Composition of Grape Pomace

GP contains significant amounts of both soluble and insoluble dietary fibers, with total dietary fiber (TDF) often ranging between 50 and 75% of the dry matter, providing benefits for gastrointestinal health and aiding in weight management by enhancing satiety [31,32,33]. In addition, GP contains proteins (8.6–12%), essential fatty acids (primarily omega-6 and omega-9), and polyphenolic compounds, particularly catechins, flavonoids, and anthocyanins. These components contribute to the antioxidant, anti-inflammatory, and metabolic health-promoting effects widely documented in functional food literature [2,6].

Polyphenols in GP are associated with reduced oxidative stress, decreased inflammation, and potential protective effects against chronic diseases, such as cardiovascular diseases, type 2 diabetes, and certain types of cancer. GP’s high antioxidant capacity protects cells from oxidative damage, supporting cellular health and potentially slowing ageing [12,25]. GP’s bioactive compounds make it particularly attractive as a nutraceutical ingredient in concentrated extract form or as a powder added to various food products.

### 4.2. Application of GP in Functional Foods

The application of grape pomace in food products prepared from animal and vegetable raw materials is shown in Table 2.

#### 4.2.1. Bakery Products

GP has been extensively researched for its use in baked goods such as bread, muffins, cakes, and cookies. Incorporating GP into these products boosts fiber content, introduces beneficial bioactive compounds, and enhances shelf life due to its antioxidant properties. For instance, GP-enriched bread and cookies have improved feelings of fullness and lower glycemic response, supporting weight management and blood sugar control [55,64].

Additionally, the polyphenols in GP contribute a mild fruity flavor that complements various baked items, enhancing their sensory appeal. Research indicates that adding GP at 5–10% levels can enhance bakery products’ texture and nutritional profile without negatively impacting their taste or consumer acceptance [5]. By increasing fiber and antioxidant levels, these products align well with the functional food market’s focus on health-promoting properties.

#### 4.2.2. Dairy and Plant-Based Yogurts

GP powder has shown promise in yoghurt and other fermented dairy or plant-based alternatives by improving antioxidant capacity and dietary fiber content. Research conducted by Tseng and Zhao [6] demonstrated that yoghurt enriched with 1% GP had high consumer acceptability, particularly regarding flavor and texture. The polyphenols in GP contribute to improved health benefits and enhance shelf life by inhibiting lipid oxidation and microbial growth, both of which are crucial for maintaining the quality of dairy products. GP can complement the nutritional value of non-dairy alternatives, such as almond- or soy-based yogurts, by further enriching them with dietary fiber and bioactive compounds. While many plant-based yogurts already contain fiber depending on their formulation, the inclusion of GP offers an additional source of functional nutrients and bioactive components that can enhance their health benefits and appeal to consumers seeking fiber-enriched products.

#### 4.2.3. Meat and Meat Analog Products

The meat industry, responding to the growing demand for healthier, functional foods, is exploring using GP in meat and meat analog products. This section discusses the functional contributions of GP to meat formulations and its potential to enhance the nutritional profile, taste, texture, and shelf life of processed meat products. Certain processed meat products have high levels of fat and sodium, which pose health risks, including cardiovascular diseases, obesity, and certain types of cancer [65,66]. Integrating GP into meat formulations reduces fat content while adding fiber, antioxidants, and essential fatty acids boosts nutritional value. For example, the fiber content in GP can significantly lower the caloric density of meat products, leading to low-calorie alternatives that support weight management efforts [17,22]. Incorporating dietary fiber into meat products improves water-holding capacity, enhancing juiciness and overall texture. Fiber helps retain moisture during cooking, reducing cooking losses and maintaining tenderness, especially in low-fat formulations.

Furthermore, the polyphenolic compounds in GP exhibit antioxidant activity that prevents lipid oxidation, extending shelf life and improving product stability [23,67]. Adding GP to meat products also influences sensory attributes such as colour, flavor, and texture. The polyphenols in GP contribute a dark hue to products, making it particularly suitable for darker meat types or meat analogs that aim to replicate the characteristics of red meat [61]. Sensory studies indicate that GP-enriched meat products typically have improved flavor profiles, featuring a mild, fruity aroma from the polyphenols that complement various seasoning blends [20,68]. However, flavor challenges may arise due to the tannins and bitter compounds naturally occurring in GP, which can result in astringency. To mitigate these off-flavors, encapsulation techniques or pairing GP with compatible spices can enhance consumer acceptability [6]. Studies have shown that sausages, patties, and processed meats enriched with GP are well received, provided that the concentration of GP is balanced to maintain desirable sensory qualities [55,64]. Furthermore, novel research results indicate that GP can enhance the nutritional profile of these products while addressing sustainability goals [30]. The increasing popularity of plant-based diets has sparked interest in utilizing GP in meat analog products.

GP’s high fiber, antioxidant, and protein content make it ideal for formulating plant-based meat substitutes with improved nutritional value. Specifically, the fiber in GP enhances the structure and texture of these analogs, enabling them to emulate the mouthfeel of traditional meat products better [69]. GP has been successfully incorporated into plant-based sausages, burgers, and meatballs, where it contributes dietary fiber and serves as a natural colorant, enhancing the visual appeal of these products. The presence of polyphenols aids in extending the shelf life of plant-based meat substitutes, which are often susceptible to oxidation and rancidity due to their high oil content. Additionally, GP’s unique flavor profile can enhance the taste of meat analogs, delivering a more satisfying experience than unflavored plant-based ingredients [5,24]. Despite GP’s many benefits for meat product formulation, some technological challenges remain. Proper processing of GP for meat applications necessitates careful control of particle size and incorporation techniques to ensure a seamless blend without disrupting the meat matrix. Larger particle sizes may adversely affect texture and consistency, resulting in a grainy or gritty mouthfeel that may be unappealing in specific meat products. Therefore, reducing particle size and achieving homogenization is crucial for producing a palatable end product [28]. Another important consideration is the stability of GP during high-temperature processing, as some antioxidants may degrade when exposed to heat. Research indicates that polyphenols can partially lose their effectiveness during cooking, making it necessary to implement techniques to stabilize these compounds, such as encapsulation or low-temperature processing [70,71]. Further research into processing methods that preserve the functional integrity of GP is essential to maximize its benefits in meat products.

#### 4.2.4. Smoothies and Beverages

GP can be easily incorporated into smoothies and other health-focused beverages as a natural source of fiber and antioxidants. Its fine powdered form can be added to smoothies, where its mild flavor enhances the fruit tastes without overpowering them. In addition to boosting fiber content, GP improves the polyphenolic profile of smoothies, contributing to their health-promoting properties and aligning with consumer interest in clean-label, nutrient-dense beverage options [70].

Smoothies enriched with GP have been shown to provide prolonged satiety and a moderate release of energy, making them suitable for weight management programs or meal replacements for those looking for low-calorie options with added health benefits. Given the growing demand for beverages with functional health claims, GP-enriched smoothies and juices present a promising market opportunity.

#### 4.2.5. Cereal and Snack Bars

As the trend for on-the-go nutrition continues to grow, snack bars made with GP (guar gum) offer a convenient, high-fiber, and antioxidant-rich option. The fiber helps promote feelings of fullness, while the antioxidants protect against oxidative stress. Additionally, GP improves the texture of snack bars, adding a slight crunch that many people prefer in cereal bars.

Research shows that incorporating GP at levels between 5% and 15% in snack bars does not negatively impact taste but enhances nutritional value [8]. Furthermore, the inclusion of GP helps extend the shelf life of these bars by preventing lipid oxidation, which is crucial for the stability of nuts and seeds commonly used in cereal varieties. GP-enriched snack bars fit well within the trends of functional and clean-label products, appealing to health-conscious consumers.

### 4.3. Potential for Grape Pomace in Nutraceutical Formulations

Nutraceuticals, which are concentrated forms of functional compounds derived from foods, effectively utilize the bioactive compounds in GP (a specific food source) for targeted health benefits. GP can be processed into extracts, powders, or encapsulated forms that deliver polyphenols, dietary fiber, and other bioactive compounds in a concentrated manner. The polyphenolic compounds in GP, particularly flavonoids and anthocyanins, offer powerful antioxidant effects that can help reduce oxidative stress. This reduction may slow the progression of age-related diseases and enhance skin health [44]. Antioxidant supplements derived from GP could be marketed as anti-ageing or skin health products, appealing to consumers wanting to maintain cellular health. However, the evidence primarily stems from in vitro and animal studies, which necessitates further investigation in human clinical trials to confirm these effects and determine practical dietary recommendations.

GP’s high dietary fiber and the role of GP-derived polyphenols in modulating gut microbiota composition and reducing inflammation have been documented [27] (Li et al., 2022). Insoluble fibers improve bowel regularity, while the prebiotic properties of GP fibers support the growth of beneficial gut bacteria. GP could be processed into fiber supplements specifically targeting digestive health and available in formats such as powders, capsules, or drink mixes. Research indicates that fiber derived from GP can promote colonic fermentation, increasing lactic acid bacteria and bifidobacteria, which are essential for maintaining gut health [15,25]. Furthermore, GP’s combination of fiber, polyphenols, and low-calorie content makes it suitable for weight management supplements. The fiber in GP promotes feelings of fullness, while the polyphenols may help regulate blood glucose levels and reduce insulin resistance, thus supporting weight management efforts.

Additionally, catechins in GP have been linked to improved fat metabolism, making GP extracts appealing for inclusion in weight-loss formulations [16]. Lastly, the polyphenols in GP, especially catechins and flavonoids, have demonstrated anti-inflammatory effects, which can benefit cardiovascular health. Studies have shown that these polyphenols can help lower levels of inflammatory markers in the body, thereby reducing the risk of chronic diseases such as cardiovascular disease [31]. Nutraceutical formulations containing GP extract could be marketed for heart health, targeting consumers at risk of or managing cardiovascular issues.

### 4.4. Challenges and Considerations in Functional Food and Nutraceutical Development

While GP offers numerous benefits as a functional ingredient, it also presents some challenges. The high tannin content and astringency associated with GP polyphenols can affect the taste and acceptability of products. Techniques such as encapsulation or pairing with complementary flavors can help mask these off-flavors, ensuring a positive consumer experience [71]. Enzymatic treatments using tannase have been shown to degrade tannins, reducing their astringency while preserving the antioxidant activity of GP extracts [72]. Additionally, fermentation with lactic acid bacteria can also mitigate bitterness and enhance the sensory profile of GP-enriched products [70]. Moreover, the bioavailability of polyphenols in GP may vary based on the extraction process and the food matrix, highlighting the need for optimization to maximize health benefits [17].

Another challenge is the stability of GP’s bioactive compounds during processing. Heat and exposure to oxygen can degrade polyphenols, making it essential to use gentle processing methods or protective packaging. Further research into stabilization techniques, such as microencapsulation or freeze-drying, could enhance the stability and effectiveness of GP in functional food and nutraceutical products.

While different studies suggest various mitigation strategies, few have explored their scalability or economic feasibility. For example, Cheng et al. [71] demonstrate the effectiveness of encapsulation, but their methodology relies on expensive materials and equipment, limiting industrial adoption. Incorporating cost–benefit analyses and pilot-scale studies into future research could address these gaps and improve the practical applicability of these solutions.

## 5. Conclusions

Grape pomace (GP), a byproduct of the wine industry, has shown potential for use as a functional ingredient in foods and nutraceuticals. Its composition, including dietary fiber, polyphenols, and proteins, presents opportunities for value-added applications. Additionally, GP contributes to environmental sustainability by transforming waste into high-value products. Incorporating GP into meat products, baked goods, dairy alternatives, and snacks has enhanced nutritional profiles, improved sensory qualities, and extended shelf life, thereby providing consumers with healthier and more sustainable options. The bioactive compounds and dietary fibers in GP make it a versatile ingredient suitable for various food types and formats. While there are challenges to overcome, such as sensory acceptance, ingredient compatibility, and the bioavailability of polyphenols, ongoing research and advancements in food processing are gradually addressing these issues.

In summary, grape pomace (GP) presents a promising opportunity to align health-focused product development with environmental sustainability. Its incorporation into food and nutraceutical products has the potential to contribute to the development of functional and nutritionally enriched offerings while addressing global challenges of food waste and sustainable resource utilization. However, its broader adoption will require addressing existing limitations, such as variability in composition, bioavailability of nutrients, and technical challenges in food processing. Future research should prioritize optimizing GP’s applications across diverse food systems, evaluating its impact on human health through robust clinical studies, and exploring scalable processing methods to fully realize its potential as a sustainable and health-promoting ingredient in an evolving food landscape.

## Figures and Tables

**Table 1:** The relative proportions of major components in GP [1,9,10].

Component	Range (% Dry Weight)	Key Compounds
Dietary fiber	50–75	Cellulose, hemicellulose, lignin
Protein	8–12	Essential amino acids
Lipids	7–12	Linoleic acid, oleic acid
Phenolic compounds	4–5	Catechins, proanthocyanidins
Minerals	1.3–3.8	Potassium, calcium, magnesium

**Table 2:** (**A**). Overview of application of grape pomace in various meat products. (**B**) Overview of application of grape pomace in other food products.

Product	GP Addition	Technological Impact	Results of Enrichment	References
(**A**)				
Chicken patties	Grape powder (1%)	pH regulator (acidifier)	Restructured chicken slices with fruit powder can be safely refrigerated for up to 20 days, maintaining quality and showing significantly lower SPC (*p* < 0.01) compared to control and BHT (200 ppm) products, except on day 6 when differences were not significant.	[34]
Chicken patties	Grape seed extract (0.1%)	pH regulator (acidifier)	Improved stability	[35]
Raw and cooked chicken meat	Wine industry residues (mixture of seeds and peels)	pH regulator (acidifier) Antioxidative effect	Cooked chicken meat with GP showed TBARS values of 1.35–2.24 mg MDA/kg, significantly lower than the control (4.75–7.71 mg MDA/kg) over 9 months of storage.	[36]
Beef sausage	1 and 2%, *w*/*w* of GP powder, with various levels of Na-nitrite (30, 60 and 120 mg/kg).	Antioxidative activity	Sausages with GP exhibited decreased lipid oxidation, altered color parameters (reduced lightness and yellowness, increased redness), and had higher sensory scores for taste and odor.	[37]
Cooked pork patties	Grape Seed Extract (GSE) (0–1000 μg/g muscle)	Antioxidant activity	GP effectively reduced the intensity of warmed-over flavor caused by oxidative rancidity, without significantly impacting sensory scores for quality attributes.	[38]
Cooked beef and pork	GSE	Antioxidative activity	Reduction in off-odors.	[39]
Low-sulphite beef patties	GSE (0–300 mg per kg of meat).	Potent antioxidant activity	Sausages containing 1% GP showed reduced lipid oxidation values. GP enhanced the preservative effects of SO_2_ on beef patties, particularly in preventing meat oxidation. The level of GP influenced the lightness, yellowness, and redness of sausages, with significant effects on total phenol content. Sausages with GP had higher taste and odor scores.	[39]
Nitrite-reduced beef sausages	Red GP (1 and 2%, *w*/*w*)	Potent antioxidant activity Antimicrobial activity	The addition of GP did not significantly affect the acceptability of beef sausages (*p* > 0.05), and these sausages received higher sensory scores. They also exhibited a lower microbial count compared to the control sausages.	[37]
Hamburger meat	GP microencapsulated	Antioxidative activity	Superior oxidation stability compared to the control, highlighting its significant potential as a natural antioxidant.	[40]
Cooked chicken hamburgers	GP	Antioxidative activity	There was a 24% reduction in lightness values, a 2.22-fold increase in redness, and a decrease in yellowness.	[41]
Restructured mutton slice	GSE	Antioxidant activity Antimicrobial activity	Better antioxidant and antimicrobial properties compared to both the control and BHA.	[42]
Raw and cooked chicken hamburgers	Grape antioxidant dietary fibers (GADF)	Antioxidant activity	Increased redness, with a reduction in lightness and yellowness, while maintaining the acceptability of HBUs. Lipid oxidation was slowed in samples with 2% GADF, and RSC values were protected in 1% GADF samples, though significantly reduced in 2% GADF samples. Overall, GADF helped retard lipid oxidation during storage.	[43]
Minced fish muscle (*Trachurus trachurus*)	GADF	Antioxidant activity	Significant changes within the first 3 months, with up to 225% increased effectiveness in FRAP and DPPH assays. Reduced formation of conjugated diene and triene hydroperoxides, a 57.28% decrease in TBA-i values, and a 62.34% inhibition of oxidation.	[44]
Minced fish muscle (*Trachurus trachurus*)	GADF	Antioxidant activity	No protection against protein aggregation was observed. Water retention increased with higher fiber levels, while thawing drip and cooking yield decreased. Shear strength, chewiness, hardness, and cohesiveness significantly dropped. The 2% GADF samples had the highest acceptability and 69.4% oxidation inhibition.	[45]
Meagre sausages	GADF	Antioxidant activity	Higher exudation and decreased texture (hardness and elasticity). Increased acidity, darkness, redness, and yellowness. Improved radical scavenging activity and reduced TBAR values. Lower sausage acceptability, but acceptable microbiological quality and reduced H2S producer counts.	[46]
(**B**)				
Yogurt and salad	Grape antioxidant dietary fibers (GADF)	Antioxidant activity and source of DF	Decrease in lightness and hue, with increased chroma, redness, and blueness compared to liquid pomace and freeze-dried GP extracts. Lower pH and increased synaeresis in yogurt with >3% wine GP, leading to reduced viscosity and higher lactic acid. No significant effect on sensory scores. Lower peroxide values and reduced total phenolic content during storage, though salad dressing had less reduction due to lower pH. Higher radical scavenging activity and significant increase in antioxidant content compared to control yogurt.	[6,47]
White bread	GPs from four cultivars (replacement of 5–10% of flour in a white bread formula)	Antioxidant activity and source of DF	Bread with 5% GP had a similar loaf volume but a darker color compared to the control, while bread with 10% GP became denser. Dietary fiber (DF), polyphenol, and antioxidant activity increased with higher GP levels. Consumer preferences for aroma, taste, and texture were significantly influenced.	[48]
Bread	GP flour	Antioxidant activity and source of DF	The addition of grape marc and olive leaves resulted in the highest phenolic content and antioxidant activity, with physical and sensory properties significantly modified by all types of polyphenolic extracts.	[49]
Wheat bread	GP flour fortification within 0, 5, and 10 g/100 g.	Antioxidant activity and source of DF	Grape pomace powder (GPP) increased the total dietary fiber (TDF) content in bread without affecting overall acceptability. Products became more tenacious and less extensible. As GPP inclusion increased, pH values and volume decreased, while total phenolic content (TPC) and antioxidant capacity, measured by FRAP and ABTS assays, rose.	[50,51]
Breadstick	Replacing wheat flour with 0.5 and 10 g 100 g^−1^ of GP flour.	Antioxidant activity and source of DF	GP flour shows promising potential for producing fiber-rich, bioactive-fortified breadsticks.	[52,53]
Pizza (innovative pizza bases)	GP in the form of a skin flour or a flour prepared from a mix of grape skin and seeds, by replacing 15, 20, and 25% of wheat flour.	Antioxidant activity and source of DF	The key aspects include a significant increase in total dietary fiber (TDF) content (>3 and >6 g/100 g), qualifying as “source of fiber” and “high fiber content” according to EC Regulation 1924/2006. Additionally, there is an increase in anthocyanin and phenolic compound content, along with enhanced antioxidant (AO) activity.	[54]
Cakes	4%, 6%, 8% and 10% GP powder (GPP)	Antioxidant activity and source of DF	The addition of 4%, 6%, 8%, and 10% GPP increased ash, lipids, proteins, fibers, phenolics, anthocyanins, total polyphenols, and antioxidant capacity. Cakes were enriched with free gallic acid, catechin, and quercetin. The cake with 4% GPP exhibited the best technological and sensory quality.	[55]
GP-enriched biscuits	GP	Antioxidant activity and source of DF	Higher TPC values and antioxidant activity against DPPH and ABTS radicals compared to control wheat biscuits.	[56]
Wheat biscuits	White grape pomace (WGP) was incorporated in wheat flour: 10, 20 and 30% (*w*/*w*)	Rheological, nutraceutical, physical and sensory properties.	Water absorption decreased from 56.4% (0% WGP) to 45.9% (30% WGP); hardness reduced, with a decline in brightness and yellowness in all enriched samples. Adding 10% WGP led to an ~88% increase in TDF content. Phenolic compounds rose from 0.11 mg g^−1^ (0% WGP) to 1.07 mg g^−1^ (30% WGP). WGP addition significantly improved antioxidant properties. Biscuits with 10% WGP were acceptable, offering a novel alternative source of dietary fiber and phenols.	[57]
Cookies	Incorporation of 2.5, 5.0, 7.5, and 10.0% of GPP with 3 different granulations (0.25, 0.50, 1.00 mm)	Sensory analysis—and consumer perception on food waste management	Reformulated cookies were well received, with good appearance, appealing colors, and pleasant aroma and taste. The highest-rated cookies were those enriched with 1.00 mm GPP granulation.	[3]
Muffins with reduced fat content and increased fiber content.	Adding 15% of GPP with different particle size fractions (600–425, 425–300, 300–212 and 212–150 µm).	Antioxidant activity and source of DF	The addition of GPP, regardless of particle size, resulted in muffins rich in antioxidant molecules and total dietary fiber (>3 g/100 g), meeting the “source of fiber” claim (EC Regulation 1924/2006). As particle size decreased, anthocyanin, TPC, and antioxidant activity (measured by ABTS and DPPH) increased. However, muffin hardness and lightness were negatively affected. The muffins exhibited good polyphenolic, dietary fiber, and antioxidant content.	[58]
Milk chocolate	Addition of 3.5% GP	Antioxidant activity and source of DF	Significantly increased the polyphenol content and AO activity	[59]
Pasta	GP (*Vitis vinifera* L., red and/or white)	Antioxidant activity	GP enhanced the volatile profile by increasing esters, terpenes, alcohols, and aldehydes compared to the control. Additionally, it boosted antioxidant activity, fiber content, and polyphenol levels in pasta.	[60]
Fettuccini pasta	Incorporation of 25, 50 and 75 g/kg of grape marc powder (GMP)	Antioxidant activity	GP increased the TPC, condensed tannins, monomeric anthocyanins, and antioxidant capacity in cooked pasta. A 25 g/kg GMP addition provided the best overall acceptance with minimal color changes.	[61]
Beverage	5% GP skin powder dissolved in water	Antioxidant activity	Grape skin pomace is a source of bioactive compounds with antioxidant and anti-inflammatory properties.	[62]
Functional cookies	GP (cv. Muscat) (6% *w*/*w* in cookie dough)	Antioxidant activity and nutritional benefit	GP-enriched cookies exhibited health benefits, including high antioxidant potential and improved shelf life.	[63]

## Data Availability

No new data were created or analyzed in this study. Data sharing is not applicable to this article.
